# Ascorbic acid alters cell fate commitment of human neural progenitors in a WNT/β-catenin/ROS signaling dependent manner

**DOI:** 10.1186/s12929-017-0385-1

**Published:** 2017-10-16

**Authors:** Tareck Rharass, Margareta Lantow, Adam Gbankoto, Dieter G. Weiss, Daniela Panáková, Stéphanie Lucas

**Affiliations:** 1 Physiopathology of Inflammatory Bone Diseases, University of the Littoral Opal Coast, F-62327 Boulogne sur Mer, France; 2 Electrochemical Signaling in Development and Disease, Max Delbrück Center for Molecular Medicine in the Helmholtz Association, D-13125 Berlin, Germany; 3 Cell Biology and Biosystems Technology, Institute of Biological Sciences, University of Rostock, D-18059 Rostock, Germany; 4 Department of Animal Physiology, Faculty of Sciences and Technics, University of Abomey-Calavi, 01, BP 526 Cotonou, Benin; 5 DZHK (German Centre for Cardiovascular Research), Partner Site Berlin, D-13125 Berlin, Germany; 6 Physiopathology of Inflammatory Bone Diseases, University of the Littoral Opal Coast, Boulevard Bassin Napoléon – Quai Masset, B.P. 120, F-62327 Boulogne sur Mer, Cédex France

**Keywords:** Ascorbic acid, Dishevelled, *N*-acetyl-*L*-cysteine, Neural progenitors, Neuronal differentiation, Nucleoredoxin, Reactive oxygen species, Redox state, WNT/β-catenin signaling

## Abstract

**Background:**

Improving the neuronal yield from in vitro cultivated neural progenitor cells (NPCs) is an essential challenge in transplantation therapy in neurological disorders. In this regard, Ascorbic acid (AA) is widely used to expand neurogenesis from NPCs in cultures although the mechanisms of its action remain unclear. Neurogenesis from NPCs is regulated by the redox-sensitive WNT/β-catenin signaling pathway. We therefore aimed to investigate how AA interacts with this pathway and potentiates neurogenesis.

**Methods:**

Effects of 200 μM AA were compared with the pro-neurogenic reagent and WNT/β-catenin signaling agonist lithium chloride (LiCl), and molecules with antioxidant activities i.e. *N*-acetyl-*L*-cysteine (NAC) and ruthenium red (RuR), in differentiating neural progenitor ReNcell VM cells. Cells were supplemented with reagents for two periods of treatment: a full period encompassing the whole differentiation process versus an early short period that is restricted to the cell fate commitment stage. Intracellular redox balance and reactive oxygen species (ROS) metabolism were examined by flow cytometry using redox and ROS sensors. Confocal microscopy was performed to assess cell viability, neuronal yield, and levels of two proteins: Nucleoredoxin (NXN) and the WNT/β-catenin signaling component Dishevelled 2 (DVL2). *TUBB3* and *MYC* gene responses were evaluated by quantitative real-time PCR. DVL2-NXN complex dissociation was measured by fluorescence resonance energy transfer (FRET).

**Results:**

In contrast to NAC which predictably exhibited an antioxidant effect, AA treatment enhanced ROS metabolism with no cytotoxic induction. Both drugs altered ROS levels only at the early stage of the differentiation as no changes were held beyond the neuronal fate commitment stage. FRET studies showed that AA treatment accelerated the redox-dependent release of the initial pool of DVL2 from its sequestration by NXN, while RuR treatment hampered the dissociation of the two proteins. Accordingly, AA increased WNT/β-catenin signaling output i.e. *MYC* mRNA level, whereas RuR attenuated it. Moreover, AA improved neurogenesis as much as LiCl as both TUBB3-positive cell yield and *TUBB3* mRNA level increased, while NAC or RuR attenuated neurogenesis. Markedly, the neurogenesis outputs between the short and the full treatment with either NAC or AA were found unchanged, supporting our model that neuronal yield is altered by events taking place at the early phase of differentiation.

**Conclusions:**

Our findings demonstrate that AA treatment elevates ROS metabolism in a non-lethal manner prior to the NPCs commitment to their neuronal fate. Such effect stimulates the redox-sensitive DVL2 activation and WNT/β-catenin signaling response that would enhance the ensuing neuronal cell differentiation.

**Electronic supplementary material:**

The online version of this article (10.1186/s12929-017-0385-1) contains supplementary material, which is available to authorized users.

## Background

In the last decades, much effort has been made to overcome issues in clinical potential application of human central nervous system (CNS) precursors for transplantation in neurological diseases [1, 2]. One potent therapy consists in the use of in vitro expanded neural progenitor cells (NPCs) [3]. Precursor cells from rat embryonic ventral mesencephalon can be expanded in vitro and differentiated into dopaminergic neurons whose transplantation in hemiparkinsonian rats results in a functional recovery [4]. In addition, the transplantation into Parkinsonian rats of human dopamine neurons derived from in vitro expanded midbrain precursors results in successful grafts [5]. The success of such therapies depends on the efficiency of in vitro generation of human dopamine neurons prior to the transplantation. Numerous reports emphasize the critical impact of a simple supplementation of the culture medium with ascorbic acid (AA); doses comparable to the physiologic extracellular levels in the brain [6] facilitate the large-scale production of dopaminergic neurons from CNS precursors ([7–18]; see Table 1). AA is an essential nutrient widely admitted as an antioxidant agent in vivo [19, 20], and playing a role as cofactor in various biochemical reactions of the cell metabolism [21]. Moreover, AA treatment not only affects CNS precursor differentiation but also enhances dopamine neuron conversion from human placenta-derived mesenchymal stem cells [22], human blood embryonic stem cells [23] or adult rat skeletal muscle-derived stem cells [24], which suggests that AA could participate in the lineage cell commitment.

**Table 1:** Overview of reports stressing that AA ameliorates the neuronal differentiation from NPC progenitors

NPC models	Differentiation factors	Enhanced effects	Refs.
Rat embryonic mesencephalic cell cultures	200 μM AA	-Glial differentiation -Neurite growth -Dopamine neurons	[7]
Mouse embryonic stem cells	200 μM AA + SHH + FGF8	-Dopamine neurons -Serotonin neurons	[8]
Long-term basic fibroblast growth factor expanded rat mesencephalic precursors	100 μM AA	-Dopamine neurons	[9]
Mouse embryonic stem cells	200 μM AA + SHH + FGF8	-Dopamine neurons	[10]
Nurr1-transfected rat embryonic cortical progenitors	200 μM AA + B27	-Dopamine neurons	[11]
Rat embryonic cortical precursors	200 μM AA	-Astrocyte differentiation -Neuronal differentiation and maturation	[12]
Mouse ventral mesencephalic precursors	200 μM AA	-Astrocyte differentiation -Dopamine neurons -Gene responses related to cell fate determination, neuron development and maturation	[13]
bFGF/SHH-stimulated rat ventral mesencephalic precursors	200 μM AA	-Dopamine neurons	[14]
Rat ventral mesencephalic precursors	200 μM AA	-Dopamine neurons -Gene responses related to dopamine neuron marker, ROS response mediators and trophic factors	[15]
Mouse embryonic stem cells	200 μM AA + SHH + FGF8b	-Dopamine neurons	[16]
Rat ventral mesencephalic precursors	100 μM AA	-Dopamine neurons -Graft survival	[17]
Long-term expanded human fetal midbrain and cortical precursor cultures	200 μM AA + low O_2_ + longer diff. time	-Neuronal yield -Dopamine neurons	[18]

*AA*: Ascorbic acid

*SHH*: Sonic hedgehog

*FGF8*: Fibroblast growth factor 8

*bFGF*: Basic fibroblast growth factor

The mechanisms by which AA enhances neuronal differentiation of CNS precursors are still unclear. AA is found to change the responsiveness of genes regulating neuronal development and maturation as well as signaling pathways implicated in cell fate determination of mammal precursors [13, 15]. Importantly, AA-mediated NPCs dopaminergic differentiation is also found accompanied with an up-regulation of gene products implicated in reactive oxygen species (ROS) detoxification; such an increase in expression of antioxidant response genes has been attributed to AA pro-oxidant ability [15]. Indeed, the crucial role of ROS in the regulation of signaling pathways in many aspects of cell physiology including NPCs differentiation is well documented [25, 26]. However, the redox properties of AA are still controversial as both pro- and antioxidant effects are reported and mostly depend on the experimental conditions and models of the studies (see for reviews [20, 21]). Although no clear evidence on AA pro-oxidant effect has been provided so far in vivo, AA antioxidant role is discussed ex vivo in rat brain slices [27, 28] and in in vitro models as cultured human lymphocytes and lung cells [29, 30] and solutions [29–32]. So far, no clear evidence that AA acts as pro- or antioxidant to favor neuronal differentiation of NPCs have been provided since direct measurements of ROS levels are missing in all the reports stressing the positive role of AA in NPCs neurogenesis (see references in Table 1). Considering the important role that AA plays in the cellular metabolism, and that ROS are known to either induce cellular damage [33] or act as messengers regulating numerous redox events in many signaling pathways [25], elucidating the mechanisms by which AA improves the neuronal differentiation of CNS precursors remains an important challenge.

To address the relationship between AA effects on the ROS levels and the neuronal differentiation of NPCs, we used the immortalized human neural progenitor ReNcell VM cells that have been extensively characterized the last decade and have proved their reliability as in vitro NPC model. These cells are reported to promptly differentiate, in vitro, into glial cells and dopaminergic neurons within 2–3 days upon growth factors removal [34], and to commit to their neuronal fate within the first day of differentiation [26, 35]. Obviously, investigating the effects of AA implies to take into account that its activities depend on two main factors as all the pharmacological drugs. First, AA activities depend on the initial dosage of the reagent; we focused our investigations on the effects of the compound at the physiological relevant dose of 200 μM [6] to stay in agreement with the concentration range used in the previous studies on cultivated CNS precursors (Table 1). Second, AA effects also depend on its administration period that influences the time required for the compound to accumulate within the cells and to initiate the mechanisms implicated with its redox activity. Accordingly, we ensured that AA redox activity takes place during the beginning of the differentiation phase by supplementing the reagent to the cells 24 h prior to the differentiation was initiated and simultaneously with the induction of the differentiation. We then compared its effects with molecules well recognized for either their pro-neurogenic or antioxidant activity.

First, to elucidate the nature and the time range of AA redox properties we monitored the kinetics of both intracellular redox balance and ROS metabolism. Kinetics were performed from the first hours of the differentiation i.e. a redox-sensitive period found to modulate the neurogenesis [26], up to 1 day i.e. after the cell fate commitment phase. Second, to verify AA effect on neurogenesis we quantified the neuronal yield and assessed the gene expression level of a specific neuronal marker. Two periods of treatment were compared to clarify whether the neurogenic effect of AA happens during the cell fate commitment stage and/or after cells committed to their fate. Thus, the cell exposure to AA was either continuous up to the third day of differentiation or interrupted after the first day. Finally, to connect the redox activity of AA with its neurogenic effect we investigated how the reagent influences the response of the WNT/β-catenin signaling pathway. Since its activation is a pre-requisite for the neuronal differentiation of ReNcell VM cells [26, 36], and since its output is modulated by ROS during the cell fate commitment period [26, 35], we hypothesized that this pathway may play a key role in AA effects. Here, our findings reveal that AA treatment results in a non-lethal pro-oxidant effect in in vitro differentiated human NPCs and that it enhances ROS metabolism strictly during the cell fate commitment period. Our data also indicate that AA treatment enhances both the WNT/β-catenin signal transduction and the neuronal differentiation. Finally, they show that AA treatment facilitates the redox-sensitive dissociation of complexes made of two proteins [26, 37]: Nucleoredoxin (NXN) that is an endogenous antioxidant ubiquitously expressed in the cells [38]; and Dishevelled segment polarity protein 2 (DVL2), an essential upstream mediator of the WNT/β-catenin pathway [39] which relays the signal further downstream once its initial inactive pool is released from sequestration by NXN [26]. Altogether, our results infer that AA treatment potentiates the neuronal fate commitment of NPCs by increasing the ROS metabolism which enhances the redox-dependent WNT/β-catenin signaling output. Accordingly, our study supports the beneficial AA supplementation at physiological doses for improving the in vitro neuronal yield of human NPCs that are aimed at cell replacement and regenerative therapies in the treatment of neuronal disorders.

## Methods

### Cell culture

Experiments were conducted in the immortalized human neural progenitor ReNcell VM cells derived from the ventral midbrain of a 10-week human fetal neural tissue (Merck Millipore, Billerica, MA, USA). Cells are cultivated in flasks pre-coated with Cultrex Mouse Laminin I (Trevigen, Gaithersburg, Germany), in proliferating medium containing DMEM/F12 medium, B27 neural cell supplement, L-glutamine, gentamycin, human basic fibroblast growth factor (all from Invitrogen, Karlsruhe, Germany), human epidermal growth factor and heparin (all from Sigma-Aldrich, Steinheim, Germany) as described previously [34, 36]. When 80% cell confluence is reached, differentiation is induced by discarding the proliferating medium followed by washing steps and replacement with differentiating medium i.e. medium without growth factors.

### Cell treatment

Proliferating and/or differentiating cells were treated with 5 μM phorbol 12-myristate 13-acetate (PMA), 200 μM ascorbic acid (AA), 5 mM *N*-acetyl-*L*-cysteine (NAC), 1 mM hydrogen peroxide (H_2_O_2_), 15 mM lithium chloride (LiCl) or 0.5 μM ruthenium red (RuR) (all from Sigma-Aldrich). Different time ranges for cell treatments were applied. H_2_O_2_ or PMA were used as positive controls for redox state and ROS detections, and were added to untreated proliferating cells for 1 h or 24 h, respectively. AA or NAC were added to untreated proliferating cells for 24 h. Then, the cell differentiation was initiated and the AA-pre-treated- and NAC-pre-treated cells were respectively exposed to AA or NAC for two periods: a long period that corresponds to a drug exposure along the 72 h of differentiation (i.e. full treatment); a shorter one that consists in a drug exposure for the first 24 h of differentiation followed by an incubation of the cells in drug-free differentiating medium for the next 48 h (i.e. short treatment). LiCl or RuR were added to untreated proliferating cells for 1 h. Then a further exposure of the cells to LiCl for 24 h or to RuR for 2 h was performed at the onset of the differentiation process, followed by an incubation of the cells in drug-free medium up to the third day of differentiation. RuR effect was also examined for a full treatment along the 72 h of differentiation.

### Flow cytometry

Intracellular redox state and ROS levels were assessed through flow cytometry using the redox indicator 5(6)-carboxy-2′,7′-dichlorodihydrofluorescein diacetate (carboxy-H_2_DCFDA) (Invitrogen) and the ROS indicator dihydrorhodamine 123 (DHR123) (Sigma-Aldrich), respectively [40]. Cells were stained with 10 μM carboxy-H_2_DCFDA for 1 h or with 1 μM DHR123 for 0.5 h at 37 °C, 5% CO_2_, in the dark. After rinsing with pre-warmed culture medium, a further incubation of the carboxy-H_2_DCFDA stained-cells for 0.5 h was performed to allow the diacetate group of the probe being hydrolyzed by cellular esterases and to render the probe sensitive to oxidation according to the manufacturer’s instructions. Cells were then placed in HBSS complemented with 14 mM HEPES and 0.9% NaCl (all from Carl Roth, Karlsruhe, Germany) prior measurements. Mean fluorescence intensities in a total of 10^4^ cells were determined in each sample using EPICS XL-MCL flow cytometer system (Beckman Coulter). An unstained cell sample was carried along as a control for auto-fluorescence. Data were analyzed for 4 independent exposure experiments measured in duplicates. Results are shown as means ± SD a.u. (arbitrary units).

### Immunocytochemistry

Cells grown on glass coverslips pre-coated with poly-D-lysine then with laminin were fixed 20 min with 4% paraformaldehyde and 4% sucrose in PBS followed by 10 min quenching with 50 mM NH_4_Cl and 5 min permeabilization with 0.2% Triton-X100 (all from Sigma-Aldrich). Non-specific binding sites were blocked 1 h with 1% gelatin (Sigma-Aldrich). Cells were incubated 1 h with the following primary antibodies: mouse anti-glial fibrillary acidic protein-Cy3-conjugate (i.e. anti-GFAP; dilution 1:400) (Sigma-Aldrich, Cat# C9205); mouse anti-tubulin, beta 3 class III-FITC-conjugate (i.e. anti-TUBB3; dilution 1:80) (Abcam, Cat# ab25770); rabbit anti-dishevelled segment polarity protein 2 (i.e. anti-DVL2; dilution 1:200) (Santa Cruz Biotechnology, Cat# sc-13974); goat anti-nucleoredoxin (i.e. anti-NXN; dilution 1:200) (Santa Cruz Biotechnology, Cat# sc-161973). When required, cells were rinsed with 0.2% gelatin and incubated 45 min with anti-rabbit Alexa Fluor 488 or anti-goat Alexa Fluor 594 secondary antibodies (all from Invitrogen; dilution 1:500); then to prevent any dissociation of the secondary antibodies an additional post-fixation step (10 min with 2% paraformaldehyde and 2% sucrose) was carried out, followed by a quenching step (5 min with 50 mM NH_4_Cl). Nuclei were stained 10 min with 2 μM Hoechst 32258 (Sigma-Aldrich). Coverslips were mounted using prolong gold antifade reagent (Invitrogen).

### Confocal microscopy and quantitative image analysis

Neuronal cell differentiation imaging was performed using LSM 710 NLO confocal microscope system (Carl Zeiss). Fluorescent images of DVL2 and NXN proteins were acquired with TCS SP2 AOBS confocal laser scanning microscope (Leica). The parameter settings (detector gain and offset, pinhole size at 1 arbitrary unit, laser power, confocal section, zoom factor, frame averaging) were kept constant for all comparative set of experiments. With these settings no photobleaching was detected after several repeated measurements on the same microscopic field. Brightness/contrast adjustments were applied equally to every pixel in the images (i.e. maximum projections), for each comparative set (e.g. control vs. treatment) using Fiji/ImageJ. Adjustments were performed on individual color channels before merging images. No change to gamma settings was applied. For neuron quantitation, TUBB3-positive cells were counted and the neuronal yield was calculated as % of the population from the ratio between TUBB3-positive cells and Hoechst-stained cells (i.e. whole cell population). For quantification of protein amounts, regions of interest were set for each cell in the images based on cell boundaries and mean fluorescence intensities were measured. The background fluorescence was subtracted for each image and values were then normalized. For each time point or treatment condition, at least 10 images per experiment were recorded for both neuron counting and protein level quantification experiments. Data are shown as means ± SD from 3 independent experiments.

### Cell viability assay

Cell density was quantified to assess cytotoxicity following the different treatments. For each treatment condition, proliferating cells were seeded on glass coverslips pre-coated with poly-D-lysine and laminin at the same cell concentration. Cell differentiation was induced up to 3 days when the cell confluency reached ~80%. Then, cells were loaded with 2 μM Hoechst 32258 for 10 min followed by 2 washing steps with PBS. Coverslips were fixed and mounted as described above and images of nuclei were acquired with LSM 710 NLO confocal microscope. Nuclei were counted using Fiji/ImageJ software to determine the residual cell density. The nuclear labelling reports the number of cells in the population. Cells were treated with AA, LiCl, RuR or NAC as aforementioned. Images were acquired for proliferating cells treated for 24 h with AA, and after 72 h of differentiation. About 10 images per experiment were captured for each condition. Data were obtained from 3 independent experiments and presented as means (% of cell viability) ± SD.

### Fluorescence resonance energy transfer (FRET)

FRET microscopy was performed to assess physical binding between DVL2 and NXN proteins. FRET was measured with the sensitized emission method [41] using TCS SP2 AOBS confocal microscope as previously described [26], and the parameter settings were kept constant throughout all experiments. Different set of cell specimens were prepared to get the fully corrected FRET signal [42]: a FRET specimen consisting in a cell sample labeled with both the donor (Alexa Fluor 488-labeling DVL2) and the acceptor (Alexa Fluor 594-labeling NXN), and from which the FRET signal can be detected; and reference specimens labeled with the donor only and the acceptor only to obtain calibration coefficients required for adequate corrections of direct acceptor excitation and donor emission bleed-through. The excitation wavelengths were set at 488 nm and 594 nm for the donor and the acceptor, respectively. The detection wavelength ranges were set at 490–550 nm for the donor to avoid leakage of acceptor fluorescence into the donor image, and at 620–700 nm for the acceptor. The acquisition method of the FRET signal included suitable sequential laser switching-on to enable selective excitation of one fluorophore, and meanwhile avoid the inappropriate excitation of the other one. Once FRET signal was acquired and corrected, the proportion of direct protein-protein association i.e. FRET efficiency (FRET_eff_), was calculated using Eq. (1) [43]:

1 $$ {\mathrm{FRET}}_{\mathrm{eff}}=\left[\mathrm{B}-\mathrm{A}\times \beta -\mathrm{C}\times \left(\gamma -\alpha \times \beta \right)\right]/\left[\mathrm{C}\times \left(1-\beta \times \delta \right)\right] $$

Where A, B and C correspond to the intensities of the donor, the FRET (i.e. acceptor indirectly excited by the donor excitation) and the acceptor (i.e. acceptor selectively excited) signals, respectively, acquired from the FRET specimen. α, γ and δ consist in the calibration factors calculated from the reference specimen labeled with the acceptor only: α = A/C, γ = B/C, δ = A/B. β is a calibration factor obtained from the specimen labeled with the donor only: β = B/A. FRET_eff_ were measured inside individual cells (n = ~50 cells per time point) for every condition. Measurements were performed for 3 independent experiments, and results are presented as means (% of FRET_eff_) ± SD.

### Quantitative real-time polymerase chain reaction (qPCR)

Total RNAs were extracted with high pure RNA isolation kit (Roche, Mannheim, Germany) according to the manufacturer’s instructions. Residual contaminating genomic DNA was digested by DNase I recombinant, RNase-free (Roche). cDNA synthesis was primed with oligo(dT)18 primers and generated from 1 μg template RNA with M-MuLV RT using first strand cDNA synthesis kit (Thermo Scientific, Karlsruhe, Germany) as following: 5 min at 25 °C, 60 min at 37 °C and 5 min at 70 °C. Real-time PCR analysis was performed by mixing 100 ng template cDNA with TaqMan gene expression master mix and following TaqMan gene expression assays (all from Applied Biosystems, Darmstadt, Germany): *MY*C (Hs00153408_m1); *TUBB3* (Hs00801390_s1); *RPL13A* (Hs04194366_g1). Contents were transferred into 96-well PCR plates (Thermo Scientific) as the final concentration of cDNA in each well was 5 ng/μl. Amplifications were performed using iQ5 real-time PCR detection system (Bio-Rad) as following: 2 min at 50 °C for activation of the Uracil-N-Glycosylase; 10 min at 95 °C for polymerase activation; 40 repeats of two-step cycling (15 s at 95 °C for denaturation and 1 min at 60 °C for annealing and extension). Relative expression values were obtained by normalizing Ct values of the tested genes in comparison with Ct values of ribosomal protein L13a (RPL13A, housekeeping gene) using the ΔCt method. Each condition was assessed from 3 independent samples in duplicate. Results are presented as fold induction means ± SD from 3 independent experiments.

### Statistics

Statistical analyses were performed using two-tailed unpaired Student’s t-test with GraphPad Prism 6. *P ≤ 0.05, significantly different compared to the control (untreated proliferating cells i.e. *t* = 0 h of differentiation). ^#^ *P* ≤ 0.05, significantly different between samples at each time point. Data are presented as means ± SD and averaged from at least 3 independent experiments.

## Results

### AA treatment increases the intracellular ROS levels strictly during a narrow time range at the onset of human NPCs differentiation

**Fig. 1:**
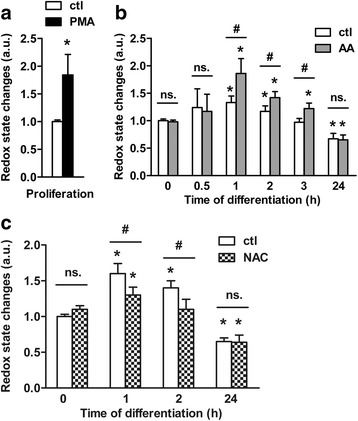
AA treatment alters the cellular redox balance at the onset of differentiation. **a** Changes in the intracellular redox state measured by flow cytometry in proliferating cells. Cells were treated with 5 μM PMA (positive control). **b** Intracellular redox state was assessed up to 24 h after the differentiation was induced. Data are compared between untreated differentiating cells and differentiating cells treated with 200 μM AA only up to 24 h post-differentiation. **c** Changes in intracellular redox state measured in untreated differentiating cells were compared with values obtained for differentiating cells treated with 5 mM NAC only up to 24 h post-differentiation. *n* = ~ 3.10^4^ cells per time point. Values are mean ± SD of three independent experiments. *P ≤ 0.05 compared with untreated differentiating cells at *t* = 0 h; ^#^ *P* ≤ 0.05 between each treatment condition at each time point; ns, non-significant

AA is reported to augment neurogenesis of mammalian CNS precursors in vitro (Table 1); yet whether it displays pro-oxidant or antioxidant effects remains uncertain. We therefore addressed this issue in differentiating ReNcell VM cells treated with the physiological dose of 200 μM of AA [6]; the cells were even pre-treated for 24 h before the induction of the differentiation to ensure that AA redox activity can take place at the onset of the differentiation process. We first examined the cellular redox balance by flow cytometry during the first 3 h of differentiation and after the cells committed to their fate (i.e. at 24 h) with the redox indicator carboxy-H_2_DCFDA [40]. Changes in the fluorescent signal level are clearly linked to the redox balance as shown with the increase triggered by the pro-oxidant reagent phorbol 12-myristate 13-acetate (PMA) [44] in proliferating cells treated for 24 h (Fig. 1a). In untreated cells, inducing the differentiation by growth factors removal led to an increased fluorescent signal from 1 h that remained stable up to 2 h and returned to baseline at 3 h (Fig. 1b). These data confirm the switch in the redox state at the early phase of human NPCs differentiation [26]. The signal even decreased below the baseline at 24 h (Fig. 1b), indicating that the physiological metabolism of the cells changed and corroborating that cells committed to different fates. Surprisingly, 24 h-pre-treatment of proliferating cells with AA did not affect the cellular redox state as the fluorescent signal stayed at baseline (Fig. 1b; *t* = 0 h). Importantly, AA treatment led to a pro-oxidant effect only during the narrow time range of the first 3 h of differentiation. The signal increased at 1 h and was 40% higher than for untreated cells (Fig. 1b; 1.9 a.u. for AA-treated cells vs. 1.3 a.u. for untreated cells). Then, the signal decreased after 3 h in AA-treated cells but still remained significantly higher than the baseline. After the cell fate commitment stage, AA did not affect the redox balance anymore as the signal at 24 h was as diminished as the one for untreated cells. Next, we assessed the effect of the potent stoichiometric ROS scavenger *N*-acetyl-*L*-cysteine (NAC) [45] on the NPCs. Untreated cells were incubated with NAC only according to a time exposure similar to AA i.e. a 24 h-pre-treatment prior to the induction of the differentiation followed by a 24 h-treatment at the onset of the differentiation. In this manner, we promoted the intracellular supply in NAC to ensure that its antioxidant effect happens during the initial differentiation phase. In contrast to AA treatment (Fig. 1b), the NAC treatment displayed an antioxidant effect as it limited the rise in the fluorescent signal occurring during the onset of differentiation when compared to control (Fig. 1c). Only a moderate increase of the signal was found at 1 h though significantly lower than for untreated cells; then the signal already returned to the baseline from 2 h. Importantly, as seen for AA treatment (Fig. 1b) NAC did not significantly modify the redox balance in 24 h-treated proliferating cells (Fig. 1c; *t* = 0 h) or after the cell fate commitment phase (Fig. 1c; *t* = 24 h). Taken together, these data support the view that AA leads to a pro-oxidant effect when supplied to human NPCs in vitro.

**Fig. 2:**
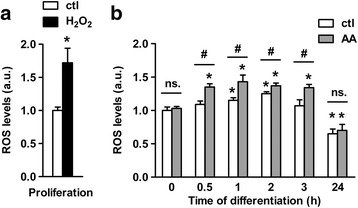
AA treatment changes the intracellular ROS metabolism at the early stage of differentiation. **a** Intracellular ROS levels has been examined by flow cytometry in proliferating cells treated with 1 mM H_2_O_2_ (positive control) for 1 h. **b** ROS levels were also assessed along the first day of differentiation. Results were compared between untreated and AA-treated differentiating cells. *n* = ~ 3.10^4^ cells per time point. Values are mean ± SD of three independent experiments. *P ≤ 0.05 compared with untreated differentiating cells at t = 0 h; ^#^ *P* ≤ 0.05 between each treatment condition at each time point; ns, non-significant

To correlate the changes in redox balance with the reactive oxygen species (ROS) metabolism, we measured the overall ROS levels following AA treatment using DHR123 [40]. Changes in the DHR123 signal reflect changes in ROS levels as an enhanced signal resulted from treatment of proliferating cells with hydrogen peroxide (H_2_O_2_) solution (Fig. 2a). The results obtained with the ROS sensor (Fig. 2b) recapitulated the data obtained through the use of the redox indicator. The fluorescent signal of DHR123 was significantly augmented upon 1 h of differentiation of untreated cells. Then it remained stable at 2 h, returned to the baseline at 3 h, and even decreased below the baseline at 24 h (Fig. 2b). AA treatment led to an earlier, higher and longer increase in ROS levels compared to untreated cells: the signal increased already from 0.5 h of differentiation, remained higher and stable up to 3 h, and decreased only later (Fig. 2b). These data clearly demonstrate that AA induces a pro-oxidant effect in vitro during the onset of the differentiation of human NPCs. Additionally, we found that AA did not affect ROS levels either during proliferation (Fig. 2b; *t* = 0 h) or after the cell fate commitment (Fig. 2b; *t* = 24 h), as the levels at these periods were comparable with the ones from untreated cells. These findings corroborate the results obtained with the redox indicator. The fact that the redox balance and the ROS levels varied only during the first hours of the differentiation upon AA or NAC treatments infers that the molecular mechanisms facilitating the redox properties of these reagents take place strictly during this critical narrow period.

.

### Pro-oxidant effect of AA treatment during the cell fate commitment phase is involved in the increase in human NPCs neurogenesis

**Fig. 3:**
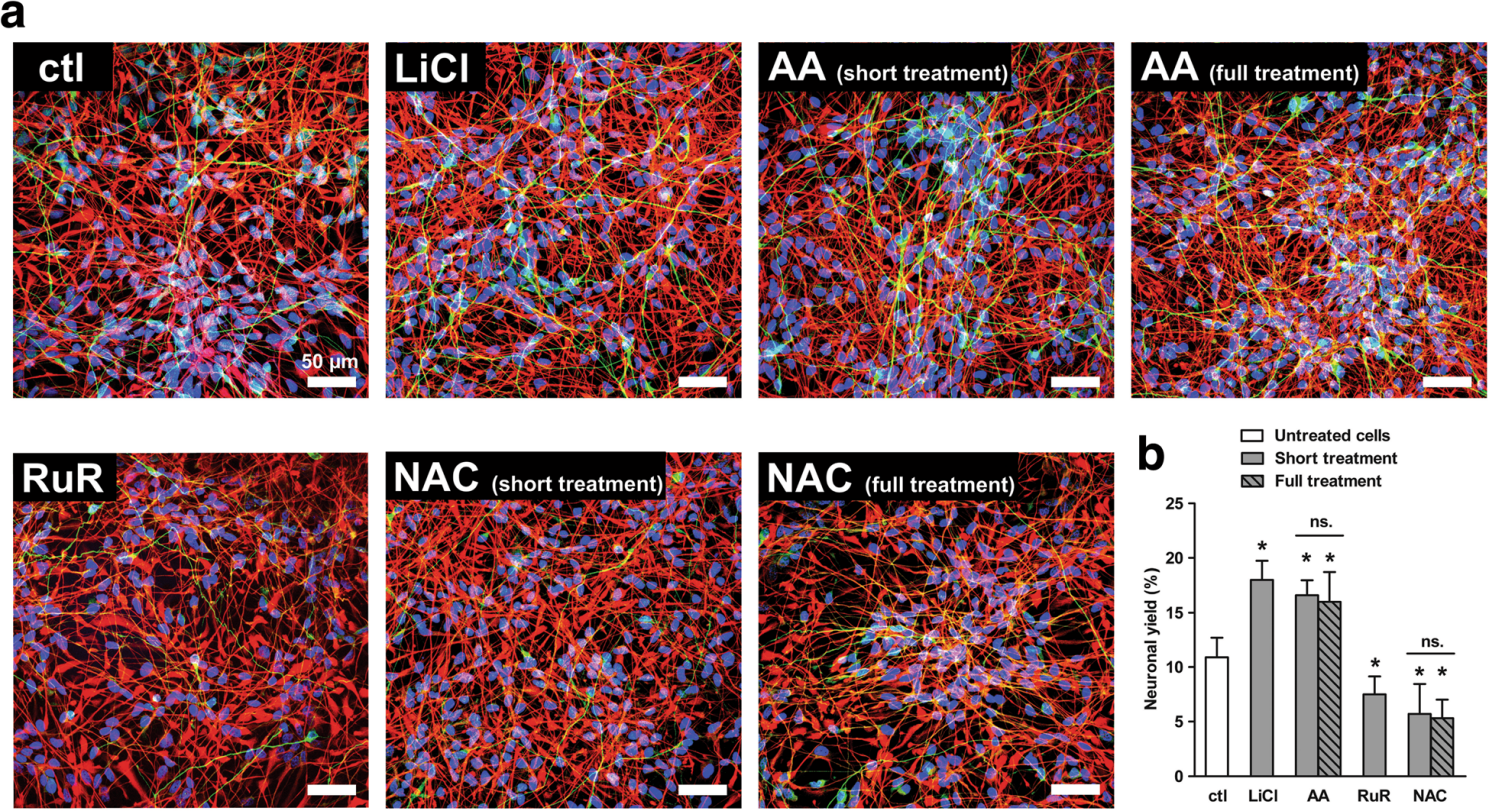
AA treatment promotes human NPC neurogenesis. **a** Confocal images of neurons (TUBB3-positive cells: green) and glial cells (GFAP-positive cells: *red*) after 72 h of differentiation. The representative image panel in the figure was selected according to a comparable cell number for each condition (*n* = 350 to 400 cells per microscopic field). Scale: 50 μm. **b** Neurons were counted and the neuronal yield was then calculated (% of the population). Data for untreated differentiated cells were compared with cells treated for two periods of incubation with 200 μM AA or 5 mM NAC (short treatment vs. full treatment), or with 15 mM LiCl or 0.5 μM RuR. *n* = ~ 3000 cells per condition. Values are mean ± SD of three independent experiments. *P ≤ 0.05 compared with untreated differentiated cells at *t* = 72 h; ^#^ *P* ≤ 0.05 between short treatment and full treatment for each condition; ns, non-significant

**Fig. 4:**
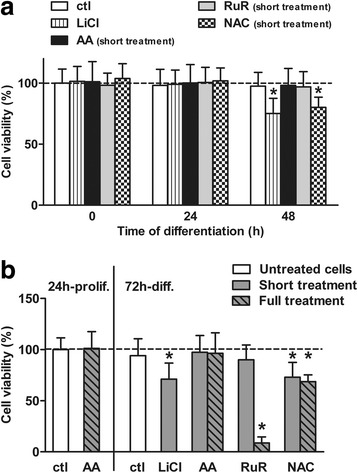
AA treatment does not affect the cell viability. **a** Cell viability was monitored at 0 h, 24 h and 48 h of differentiation. Cells were seeded at the same concentration prior to any treatment or the induction of differentiation. Prior to the differentiation was initiated, cells were pre-treated for 1 h with 15 mM LiCl or 0.5 μM RuR, and for 24 h with 200 μM AA or 5 mM NAC. A further exposure of the cells with each reagent was performed from the induction of the differentiation: up to the 24 h post-differentiation (i.e. short treatment) for LiCl, AA and NAC, and up to 2 h for RuR. Nuclei were then stained as the nuclear labelling reports the number of cells in the population. Confocal images of nuclei were acquired, and nuclei were further counted to determine the remaining cell density and to calculate the cell viability. *n* = ~ 500 cells per time point and condition. Values are mean ± SD of three independent experiments. *P ≤ 0.05 compared with untreated differentiating cells at *t* = 0 h. **b** Cell viability was assessed in proliferating or 72 h-differentiated cells for various treatments. Proliferating cells were treated for 24 h with 200 μM AA. Alternatively, differentiation was induced up to 72 h, concomitantly with distinct drug treatments: 15 mM LiCl, 200 μM AA, 5 mM NAC or 0.5 μM RuR for two periods of incubation (short treatment vs. full treatment). *n* = ~ 3000 cells per condition. Values are mean ± SD of three independent experiments. *P ≤ 0.05 compared with untreated proliferating cells

As AA showed a pro-oxidant effect in vitro at the onset of human NPCs differentiation, we wondered whether such an effect could positively or negatively affect the neurogenesis output. We thus examined by confocal microscopy the cell population labelled with the specific neuronal marker Tubulin, Beta 3 Class III (TUBB3) [46] 3 days after the differentiation was induced (Fig. 3a). The neuronal yield was quantified by counting TUBB3-positive cells (Fig. 3b). Around 11% of the untreated cell population differentiated into neurons after 3 days (Fig. 3a&b) in accordance with previous report [26]. The differentiation process did not lead to any significant cell death as examined through the cell viability assay (Fig. 4a&b). To demonstrate that cell response to early stimuli (i.e. during the onset of differentiation) drives the changes in the neuronal yield several days later, we treated the cells with lithium chloride (LiCl). LiCl is a potent glycogen synthase kinase 3 beta inhibitor [47], which has been previously reported to enhance WNT/β-catenin-mediated neurogenesis from ReNcell VM cells, and so the neuronal yield [26]. Cells were treated with LiCl 1 h prior to the differentiation was initiated, followed by a 24 h-treatment from the induction of the differentiation. As anticipated LiCl treatment enhanced the neuronal amount up to 70% after 3 days of differentiation (Fig. 3a&b; 18% of neurons). Moreover, no cytotoxicity was observed up to 24 h of differentiation i.e. during the cell fate commitment stage (Fig. 4a); the cell viability was however affected later though the reagent was not exposed to the cells anymore (Fig. 4a&b; ~25% and ~30% of dead cells at 48 h and 72 h, respectively). Next, we monitored neurogenesis ensuing the treatment of the cells with AA for 48 h, i.e. 24 h prior to the differentiation was initiated and during the first 24 h at the onset of the differentiation. Then, the cells were deprived of AA for the following 48 h (Fig. 3a; see AA short treatment). We found that such treatment led to a pro-neurogenic effect as effective as LiCl (Fig. 3a&b; 16% vs. 18% of neurons, respectively) and in a non-lethal manner (Fig. 4a&b; AA short treatment). Moreover, AA pre-treatment of the proliferating cells for 24 h did not affect the cell number that remained comparable with untreated proliferating cells (Fig. 4b). We then extended the treatment period with AA up to the third day of differentiation, i.e. 96 h-treatment in total (Fig. 3a; see AA full treatment). Interestingly, the full treatment resulted in a neuronal yield similar to the one obtained during the short treatment (Fig. 3b; 16% of neurons for both short and full treatments), and yet without inducing any cytotoxicity (Fig. 4b). Hence, AA enhances the neuronal yield without displaying any effects on the cell viability, and the pro-neurogenic mechanisms of AA happen only during the early period of the differentiation process.

We also quantified the neuronal yield resulting from cell treatment with compounds displaying antioxidant effects. First, we used ruthenium red (RuR); as we previously reported it reduces the Ca^2+^-mediated ROS metabolism i.e. ROS levels, by reversibly inhibiting the mitochondrial calcium uniporter [26]. Since a full treatment with RuR is highly cytotoxic (Fig. 4b; ~95% of dead cells), we performed a short treatment at the onset of differentiation (i.e. 3 h-treatment in total) which did not affect the cell viability as measured daily up to the third day of differentiation (Fig. 4a&b). RuR treatment reduced the neuronal amount (Fig. 3a) by 30% (Fig. 3b; 7% of neurons). Second, cells were treated with NAC only in the same way than AA: 24 h-pre-treatment followed by either a 24 h- or 72 h-treatment upon the differentiation. Importantly, both short and full treatments with NAC similarly inhibited the neurogenesis (Fig. 3a&b; ~6% of neurons at 72 h). NAC treatment did not induce any cytotoxic effect during the cell fate commitment phase (Fig. 4a; see at 24 h); yet both treatment periods led to a comparable extent in cell death later (Fig. 4a&b; ~30% of dead cells at 72 h). These results imply that extending NAC treatment beyond the cell fate commitment period does not have any supplemental inhibitory effect on neurogenesis. Furthermore, both RuR and NAC display a reverse pattern to AA: they exert antioxidant activities (see here Fig. 1b, and [26]) and they negatively modulate the neurogenesis. Therefore, the data stress the pivotal role of redox events at the early phase of differentiation in the neuronal output of human NPCs.

**Fig. 5:**
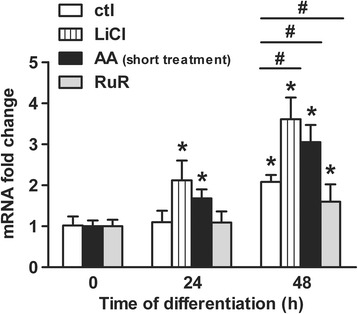
AA treatment enhances the *TUBB3* gene response. *TUBB3* mRNA levels (fold change) were analysed by quantitative real-time PCR at 0, 24 and 48 h after the differentiation was initiated. Results for untreated cells were compared with cells treated with 15 mM LiCl, 200 μM AA (short treatment) or 0.5 μM RuR. Values are mean ± SD of three independent experiments. *P ≤ 0.05 compared with untreated differentiating cells at *t* = 0 h; ^#^ *P* ≤ 0.05 between each treatment condition at *t* = 48 h; ns, non-significant

To substantiate the findings obtained by microscopy, we further examined by quantitative real-time PCR the responsiveness of the neuronal marker *TUBB3* gene in a shorter differentiation time scale i.e. at 24 h and 48 h of differentiation (Fig. 5). All treatments did not exceed the first day of differentiation to ensure that any changes in the neuronal output are connected to perturbations during the neuronal fate commitment stage only. Once the differentiation was induced by withdrawing growth factors, *TUBB3* mRNA level was up-regulated at 48 h (Fig. 5; 2.0-fold increase for control) confirming that cells undergo neuronal differentiation. As positive control, 24 h-exposure of the cells to the pro-neurogenic factor LiCl [26] already enhanced *TUBB3* mRNA level from 24 h (Fig. 5; 2.0-fold increase) to reach a 3.5-fold increase at 48 h. In line with the microscopy data, the short treatment with AA up-regulated *TUBB3* gene response in a comparable manner with LiCl: the mRNA level steadily increased by 1.7-fold already at 24 h and by 3-fold at 48 h (Fig. 5). Conversely, 3 h-treatment of the cells with the ROS metabolism inhibitor RuR at the onset of the differentiation prevented the rise in *TUBB3* mRNA level by half at 48 h when compared with untreated cells (Fig. 5; 1.5-fold vs. 2.0-fold increase, respectively). Therefore, our data support that the pro-oxidant effect of AA is instrumental during the cell fate commitment phase for improving the neuronal differentiation of human NPCs.

### AA treatment stimulates the WNT/β-catenin signal transduction and the redox-sensitive DVL2-NXN complex dissociation during the cell fate commitment phase

**Fig. 6:**
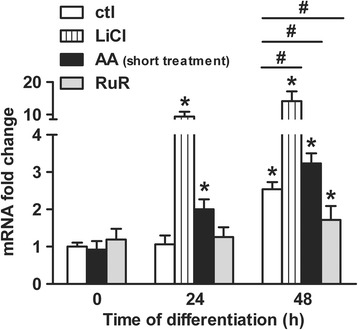
AA treatment stimulates the expression of the WNT/β-catenin signaling target gene *MYC*. *MYC* mRNA levels (fold change) were evaluated by quantitative real-time PCR at 0, 24 and 48 h after the differentiation was induced. Data for untreated cells were compared with cells treated with 15 mM LiCl, 200 μM AA (short treatment) or 0.5 μM RuR. Values are mean ± SD of three independent experiments. *P ≤ 0.05 compared with untreated differentiating cells at *t* = 0 h; ^#^ *P* ≤ 0.05 between each treatment condition at *t* = 48 h; ns, non-significant

The neuronal commitment of human NPCs depends on the WNT/β-catenin signaling output that can be regulated from the first hours of differentiation by redox-sensitive events [26]. We therefore wondered whether AA pro-oxidant effect at the onset of the differentiation may alter the response of WNT/β-catenin pathway target genes. We then assessed the expression level of v-myc avian myelocytomatosis viral oncogene homolog (*MYC*) gene [48]. Since *MYC* expression has been reported to regulate the neuronal differentiation process of ReNcell VM cells [36], its expression level reflects both the neurogenesis and the WNT/β-catenin pathway outputs. Consistent with previous report [36], we found an up-regulation of *MYC* mRNA level 48 h after the differentiation was induced in untreated cells (Fig. 6; 2.5-fold increase). Moreover, incubating the cells along the first day of differentiation with the WNT/β-catenin pathway stimulator LiCl [47] predictably enhanced *MYC* gene response to high levels at both 24 h and 48 h post-differentiation (Fig. 6; 10-fold and 15-fold increases, respectively). Importantly, exposure of the cells to AA up to the first day of differentiation (i.e. short treatment) also led to an up-regulation of *MYC* gene expression. A significant increase of the mRNA level up to 2-fold was found at 24 h and to 3-fold at 48 h (Fig. 6). In contrast, treatment with RuR limited the rise in *MYC* gene response as the mRNA level increased only to 1.7-fold at 48 h (Fig. 6). Altogether, these findings support the idea that the pro-neurogenic effect of AA is connected to both the pro-oxidant effect of the reagent during the neuronal fate commitment phase and the stimulation of early redox-dependent events altering WNT/β-catenin signaling response.

**Fig. 7:**
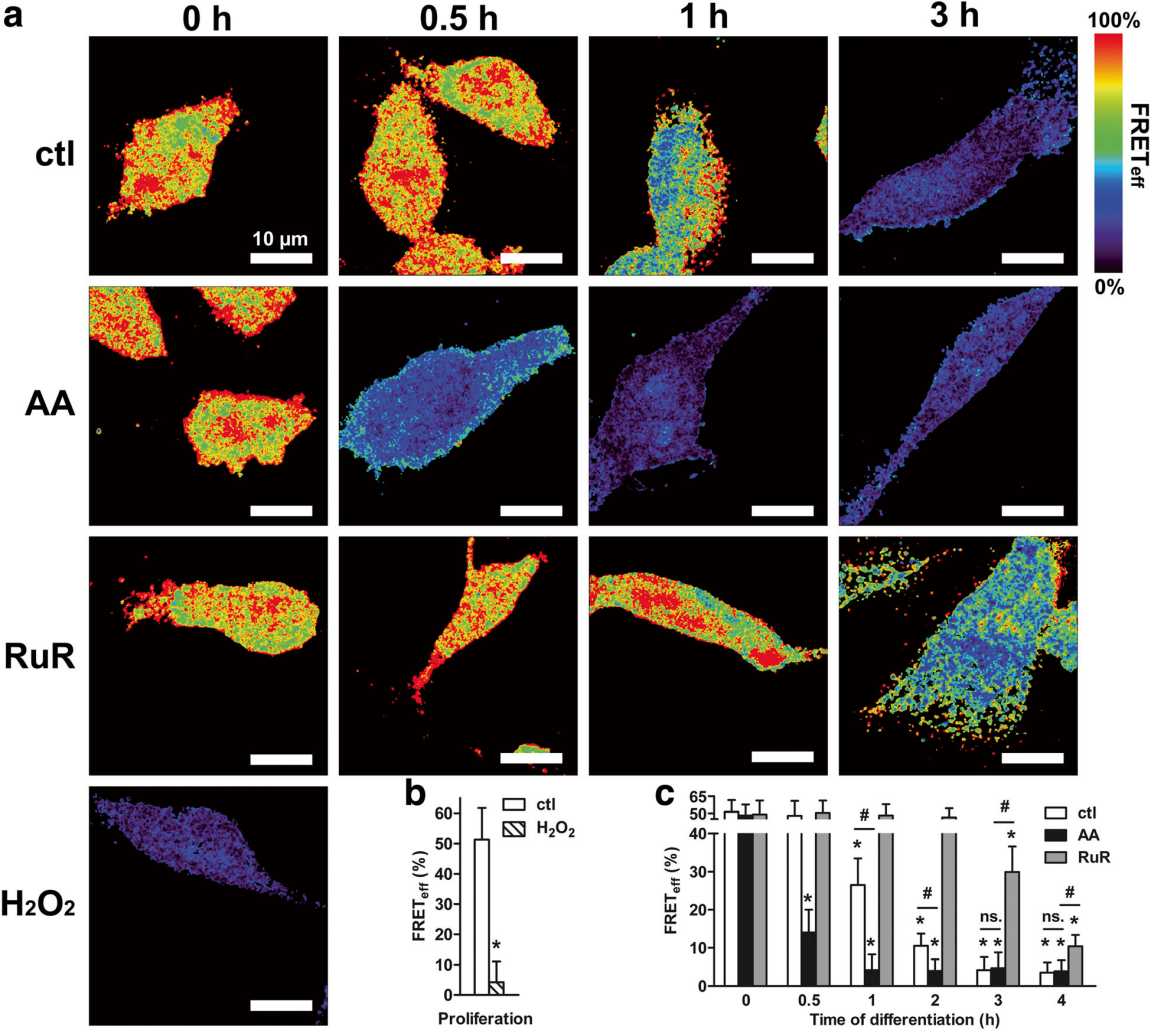
Elevated ROS metabolism mediated by AA treatment facilitates DVL2-NXN complex dissociation. **a** Representative pseudocolor images of FRET_eff_ between DVL2 and NXN proteins obtained at 0 h, 0.5 h, 1 h and 3 h after the differentiation was initiated. Images were acquired in untreated differentiating cells and compared with proliferating cells treated with 1 mM H_2_O_2_, and with differentiating cells treated with 200 μM AA or 0.5 μM RuR. The colour scale at the right of the panel is shown in %. Scale: 10 μm. **b** FRET_eff_ values were averaged and compared between untreated- and H_2_O_2_-treated proliferating cells. **c** Mean values of FRET_eff_ were also calculated at 0 h, 0.5 h, 1 h, 2 h, 3 h and 4 h of differentiation for untreated- and AA- or RuR-treated differentiating cells (*n* = ~ 50 cells per time point and condition). Values are means (% of FRET_eff_) ± SD of three independent experiments. *P ≤ 0.05 compared with untreated differentiating cells at *t* = 0 h; ^#^ *P* ≤ 0.05 each treatment condition at each time point; ns, non-significant

Nucleoredoxin (NXN) is an endogenous antioxidant [38] sequestering the WNT/β-catenin signaling key component Dishevelled segment polarity protein 2 (DVL2) [37, 39]. The release of the initial pool of DVL2 at the early stage of the differentiation is a redox-sensitive step required for relaying the WNT signal to downstream effectors [26]. Accordingly, we examined whether the early changes in ROS metabolism resulting from AA treatment may impact on DVL2 release from NXN. DVL2-NXN complex dissociation was measured by fluorescence resonance energy transfer (FRET) microscopy (Fig. 7a), and FRET efficiencies (FRET_eff_) were quantified in proliferating cells and during the first 4 h of differentiation (Fig. 7b&c). To confirm that changes in FRET_eff_ solely depend in ROS levels, we treated proliferating cells for 1 h with 1 mM H_2_O_2_ to enhance the intracellular ROS concentration. FRET_eff_ strongly decreased to 10-fold compared to untreated proliferating cells (Fig. 7a&b; ctl vs. H_2_O_2_), indicating a DVL2-NXN complex dissociation. Furthermore, we substantiated that FRET_eff_ reduction did not result from any diminishing protein quantities since both DVL2 and NXN amounts increased after H_2_O_2_ treatment as quantified by fluorescence imaging (Additional file 1: Figure S1). We then monitored FRET_eff_ during the first hours of differentiation of untreated cells. FRET_eff_ significantly diminished by 1.9-fold at 1 h of differentiation and by 5-fold at 2 h (Fig. 7a&c). After 2 h, FRET_eff_ values remained low (Fig. 7a&c; ~10-fold decrease at 3 h and 4 h). Although variations of both DVL2 and NXN amounts were held during this narrow time range (Additional file 1: Figure S1), they are not correlated with the changes in FRET_eff_, and so they did not interfere with FRET_eff_ values: from 1 h to 2 h of differentiation both proteins showed elevated levels while FRET_eff_ values decreased; the next 2 h the protein amounts returned to baseline while FRET_eff_ remained unchanged. In accordance with our previous study [26], the reduction in FRET_eff_ values is instead temporally correlated with the rise in ROS metabolism observed at the early stage of differentiation (see Fig. 2b): FRET_eff_ started to decrease at 1 h when ROS levels significantly increased, and the strongest reduction of the FRET_eff_ value happened at 3 h when the highest levels of ROS were reached and maintained in a plateau. Though ROS levels returned at baseline at 4 h, FRET_eff_ still remained at its lowest value indicating that both proteins do not reassociate anymore; the cells do no longer maintain high ROS metabolism for promoting DVL2 release as the protein complexes were already fully dissociated [26].

Next, we examined whether AA treatment alters the redox-sensitive DVL2-NXN dissociation. Strikingly, we found that changes in FRET_eff_ correlates with the variations in ROS metabolism mediated by the treatment. Neither ROS levels (see Fig. 2b) nor FRET_eff_ varied in spite of the 24 h-pre-treatment with AA prior to the induction of differentiation (Fig. 7a&c; *t* = 0 h, ctl vs. AA). However, FRET_eff_ decreased by 3.3 fold from 0.5 h of differentiation i.e. simultaneously with the beginning of the rise in ROS metabolism resulting from AA treatment; a maximum FRET_eff_ reduction by ~10 fold was already reached at 1 h (Fig. 7a&c). Here again, the FRET_eff_ reduction did not result from any diminution in the protein amounts as they rather increased at 0.5 h (Additional file 1: Figure S1). Therefore, AA treatment accelerated the DVL2-NXN complex dissociation just as it did for ROS production. Conversely, inhibiting ROS metabolism prevented the complex dissociation as shown in RuR-treated cells (Fig. 7a&c; ctl vs. RuR). FRET_eff_ remained unaltered up to 2 h of differentiation; it started to decrease only from 3 h (Fig. 7a&c; 1.6 fold-reduction) i.e. when RuR antioxidant effect was alleviated since we stopped the cell exposure to the drug [26]. As the protein quantities augmented at 3 h instead of being diminished (Additional file 1: Figure S1), it is unlikely that protein amounts interfered with FRET_eff_ values. Changes in FRET_eff_ can solely be ascribed to the variations in ROS metabolism mediated by each treatment. Accordingly, these data corroborate that the release of DVL2 from its complex with NXN is positively regulated by ROS levels. Moreover, they show that the elevated ROS metabolism mediated by AA treatment prior to the cell fate decision facilitates the release of the initial protein pool of the WNT/β-catenin signaling mediator DVL2.

## Discussion

In this study, we aimed to unravel whether and how AA redox property is involved in the well recognized pro-neurogenic effect of the reagent when supplemented at physiological doses to NPCs. Consistent with previous reports from mouse and rat progenitors [7–17], we show here that treating differentiating human NPCs (i.e. ReNcell VM cells) with AA at a physiological dose [6] stimulates their neurogenesis in vitro (Fig. 3 and 5). Yet evidence of AA effect on redox state during neuronal differentiation of NPCs have been lacking so far [7–18]. Our study provides direct measures of intracellular ROS levels (Fig. 1 and 2) and reveals that the positive regulation of the neurogenesis of human NPCs by AA is accompanied by a pro-oxidant effect mediated by the reagent during the cell fate commitment phase. Although our findings contrast with the in vivo AA antioxidant activity, one has to emphasize that many investigations support a pro-oxidant effect ex vivo [27, 28] and in vitro [29–32]. Furthermore, a few studies performed in cultured rodent progenitor cells corroborate that AA acts as pro-oxidant. First, Yu DH, et al., [15] showed that AA-mediated dopaminergic differentiation of rat mesencephalic precursors is accompanied with an up-regulation of gene products that protect against oxidative damages. Although no direct assessment of ROS levels has been performed, the authors attributed the rise in the antioxidant defense to a cellular response to AA pro-oxidant properties. Second, Bartsch C, et al., [49] reported that AA treatment of mouse embryonic stem cells increases ROS levels and stimulates the cardiomyogenesis.

Furthermore, we report that the positive regulation of the neuronal differentiation of human NPCs by AA is independent of any effects on cell viability. The effect of AA treatment on NPCs neurogenesis and viability was compared to the treatments with the pro-neurogenic reagent LiCl [26] and the antioxidant NAC [45]. Several studies emphasized the therapeutical potential of LiCl in cell replacement therapies for CNS regeneration [50–52]. Our data corroborate these previous reports; LiCl enhances neurogenesis in our cell model as shown by the increases of both the expression of the neuronal marker *TUBB3* gene from 24 h (Fig. 5) and the neuronal amount quantified at 72 h by microscopy (Fig. 3). LiCl treatment did not display any cytotoxic effect during the cell fate commitment period i.e. the first 24 h; yet it led to cell death later (Fig. 4). Of note, LiCl cytotoxicity has been reported for comparable dose ranges and exposition times of the drug in various cell types [53, 54]. Moreover, it is unlikely that the increased quantity of neurons measured at 3 days following the treatment mainly resulted from a toxicity affecting selectively the non-neuronal cells; it has been reported that cytotoxic doses of LiCl impaired without distinction both neuronal and glial cell subpopulations differentiated from rat neural progenitors [50]. As for NAC, it led to a late cytotoxicity comparable with LiCl although the antioxidant induced an opposite effect on neurogenesis. Such a cytotoxic effect of the reagent is known for long-term treatments with millimolar doses [55–57]. Conversely, AA enhances the neuronal yield as much as LiCl but in a non-lethal manner, though these findings contrast with studies reporting that AA promotes cytotoxicity in vitro [27, 28, 58]. Given that AA treatment increases ROS metabolism, one could have anticipated a rise in cell damage and death resulting from an excessive ROS production [33]. However, whatever the exposure duration (short- vs. long treatment) the physiological dose of 200 μM of AA is not cytotoxic in our study, making it unlikely that AA mediated a ROS generation sufficient to induce an oxidative stress. One could then wonder whether the non-lethal moderate increase in the intracellular production of ROS mediated by AA treatment may rather act as messengers regulating redox-dependent events [25] which positively influence human NPCs neurogenesis.

Our earlier investigations in ReNcell VM cells revealed that the first hours of differentiation are crucial in the regulation of the neuronal output as this early period happens prior to the neuronal fate commitment [26, 35]. In addition, we previously demonstrated that changes in ROS metabolic cues are endogenously induced during a tight timing occurring right from the first hours upon the initiation of neuronal differentiation of the cells [26, 35]. The physiologic switch in ROS metabolism was found to modulate the response amplitude of the WNT/β-catenin pathway via the control of the activity of the redox-sensitive target NXN, an ubiquitous antioxidant [38] that sequestrates the WNT/β-catenin signaling downstream effector DVL2 [26]. In ReNcell VM cells the WNT/β-catenin pathway has been found to regulate the neuronal differentiation [36], and the early modulation of this pathway by ROS has been shown to further alter the neuronal commitment of the cells [26]. Remarkably, our current data unravel that AA pro-oxidant effect strictly takes place during the first hours of differentiation. Moreover, it has been reported that AA treatment down-regulates the expression of the glycogen synthase kinase 3 beta gene during neuronal differentiation of rat precursors [15] suggesting that AA may influence WNT/β-catenin pathway output. Taking all of these observations together, we reasoned that both AA pro-neurogenic and pro-oxidant effects may be connected together through the stimulation of the redox-sensitive WNT/β-catenin pathway, which subsequently could alter the neuronal differentiation of human NPCs. Our current findings show that the inhibition of ROS metabolism by antioxidants reduces both the WNT/β-catenin signaling output i.e. *MYC* gene expression (Fig. 6) and the neurogenesis (Fig. 3 and 5). Conversely, AA enhances the WNT/β-catenin signaling response and the neuronal yield. Importantly, one has to point out that changes in the magnitudes of both the WNT/β-catenin signaling response and the neurogenesis are solely linked to the alterations of the ROS metabolism prior to the neuronal fate commitment. First, beyond the cell fate commitment phase (i.e. at 24-h post-differentiation), neither NAC nor AA treatments affect the intracellular ROS metabolism as the levels are similar to untreated differentiated cells. Second, full treatments (i.e. up to 72-h post-differentiation) with either NAC or AA modify the neuronal yields similarly to short treatments (i.e. up to 24-h post-differentiation).

By which mechanism the ROS production mediated by AA treatment may interfere with the WNT/β-catenin pathway and so the neurogenesis? Interestingly, our data indicate that the variations in the WNT/β-catenin signaling outputs are related to the extent in the ROS-dependent dissociation of DVL2-NXN complexes at the beginning of the differentiation process. We previously reported that during the proliferation of human NPCs an initial pool of DVL2 is kept inactive as the proteins bind to NXN molecules [26]. Once ROS metabolism increases at the onset of the differentiation, DVL2 proteins dissociate from NXN, and thus are activated and recruited in the signaling cascade for relaying downstream the signal [26]. In line with this previous report, we corroborate by FRET microscopy that the protein complex dissociation is temporally correlated to ROS metabolism at the onset of the human NPCs differentiation (compare Fig. 2 vs Fig. 7). In addition, we demonstrate that the extent of DVL2 release is regulated by the amplitude and the promptness of the ROS production: a delayed and low DVL2 dissociation resulted from the inhibition of mitochondrial ROS formation mediated by the early and punctual RuR treatment (Fig. 7 and [26]); in contrast, a strong DVL2 release followed the treatment of proliferating cells with the pro-oxidant reagent H_2_O_2_ (Fig. 7 and [37]). As for AA, we reveal that the initial inactive pool of sequestered DVL2 promptly dissociates as a result of the early and high increase in ROS metabolism following the treatment. The dissociation of the totality of the protein complexes happens quickly in response to the maintaining of high ROS levels. Next, the proteins do not form complexes anymore despite their intracellular accumulation and further de novo synthesis (Additional file 1: Figure S1 and [26]); NXN are rather implicated in detoxifying ROS. No protein re-association occurs even after the ROS metabolism returned to baseline. Once released, DVL2 proteins become active and commit to the WNT/β-catenin signaling cascade [26]. DVL2 release tunes the extent of the WNT/β-catenin signal transduction; in other words, the earlier the initial pool of DVL2 is released and activated during the cell fate commitment stage, the more efficient and sustained is the signal [26]. Accordingly, the elevated ROS metabolism mediated by AA treatment prior to the cell fate decision facilitates DVL2 activation, and so ameliorates the efficiency of the WNT/β-catenin signal transduction; this leads to an enhanced expression of target genes e.g. *MYC* and *TUBB3* [36], that finally results in an augmented neuronal yield.

The molecular mechanisms by which AA increases intracellular ROS formation in our in vitro human NPCs model have not been thoroughly investigated here. In this study, we focused on the concentration of 200 μM since this physiological dose has been mostly used in the reports emphasizing a positive role of AA in NPCs neurogenesis; one should therefore cautiously consider that the mechanisms implicated in AA-mediated ROS depend on the dose of the compound. One could however suggest a mechanism involving redox reactions resulting from AA conversion to substrates. It has been reported that AA pro-oxidant effect results from its extracellular oxidation to dehydroascorbate that is transported in the cells and further reduced back to ascorbate [59]. However, since these reactions also lead to cell death [27, 28] such a mechanism may be unlikely in ReNcell VM cells, as AA did not alter their viability. Another conceivable mechanism may implicate AA interaction with transition metal ions although conflicting studies are reported. First, such interactions either increase [31, 32] or decrease the ROS production [20]. Next, both pro- and antioxidant effects happen simultaneously but in distinct compartments, i.e. respectively in intracellular and extracellular compartments [29]. Finally, AA-mediated ROS production is found independent from any interactions with transition metal ions [27, 28]. Thus, further studies are needed to elucidate how physiological doses of AA mediate ROS generation in our cells. As this was observed only during the cell fate commitment period, one could suggest that the molecular machinery required for this process may depend on the cellular differentiation and metabolic stage of the NPCs. Such molecular machinery may be efficient in cells at the early stage of differentiation, but neither during the proliferation stage nor in partially or terminally differentiated cells.

To date, besides the pro-oxidant effect of AA treatment demonstrated in this study, a few more potential mechanisms have been previously proposed to explain AA pro-neurogenic effects. Considering findings indicating that AA protects cells from apoptosis [60] and that dopamine auto-oxidation causes neuronal damages [58], one could speculate that AA antioxidant property might protect neurons from dopamine-mediated cytotoxic effect. Such a hypothesis is however questionable. First, many investigations challenge AA antioxidant properties [27–32]. Second, AA is also reported either potentiating the dopamine-mediated neurotoxicity [58] or failing to expand dopaminergic differentiation at doses preventing a potential dopamine auto-oxidation [9]. A more likely additional mechanism that can be attributed to AA treatment involves the stimulation of the extracellular matrix assembly needed for the development of nervous system [61]. Such mechanism is consistent with findings from Shin DM, et al., [13] showing that AA treatment up-regulates the expression of genes encoding for the extracellular membrane-associated protein pleiotrophin and the extracellular matrix glycoprotein vitronectin during the neuronal differentiation of mouse precursors. Furthermore, Yu DH, et al., [15] report an up-regulation of the expression levels of procollagen genes in neurons differentiated from cultured rat precursors treated with AA. Therefore, we do not exclude that AA pro-neurogenic effect in human NPCs can rely on other activities of the reagent in addition to its pro-oxidant effect. Actually, it is likely that both AA mechanisms i.e. ROS-mediated signaling and extracellular matrix remodeling, can act in concert to positively modulate NPCs neurogenesis as it is well-accepted that extracellular matrix interacts with signaling pathways [62].

## Conclusions

Our study reveals that the beneficial role of AA treatment in the neuronal differentiation of human NPCs results from its non-lethal pro-oxidant effect. Moreover, our findings are consistent with studies performed in rodent precursors showing that AA stimulates expression of genes involved in neuronal development and maturation, as well as gene responsiveness for products implicated in signaling pathways that control the neuronal cell fate determination [13, 15]. Here, we demonstrate in human NPCs that increased ROS metabolic cues following AA treatment are strictly anchored to a period prior to the neuronal fate commitment. Such elevated ROS metabolism promotes DVL2 activation by facilitating the dissociation of the initial inactivated pool of the proteins from NXN sequestration. As a result, the WNT/β-catenin signal transduction is ameliorated and enhances neurogenic gene expression that stimulates the decision of the cells to commit to their neuronal fate. Our findings also point out the substantial role of the redox balance in in vitro cultured NPCs for improving the neuronal yield necessary for transplantation in regenerative therapies. Finally, they emphasize the importance to control carefully the experimental conditions including the compounds in the cell culture media as previously suggested [63].

## Additional files

Additional file 1: Figure S1. Changes in DVL2 and NXN protein amounts do not correlate with variations in FRET_eff_. Confocal images of DVL2 (**a**) and NXN proteins (**b**) were acquired in proliferating and differentiating cells treated or not with 200 μM AA, 0.5 μM RuR or 1 mM H_2_O_2_. Mean fluorescence intensities were then quantified at 0 h, 0.5 h, 1 h, 2 h, 3 h and 4 h of differentiation for each protein (see respective bar graphs). These data demonstrate that decreases in FRET_eff_ values (see Fig. 7) do not result from any reduction in protein amounts which rather increase when DVL2-NXN complexes begin to dissociate. Scale: 10 μm. *n* = ~ 50 cells per time point and condition. Values are means ± SD of three independent experiments. *P ≤ 0.05 compared with untreated differentiating cells at *t* = 0 h; ^#^ *P* ≤ 0.05 each treatment condition at each time point; ns, non-significant. (JPEG 5532 kb)

## Abbreviations

AA: Ascorbic acid
Carboxy-H_2_DCFDA: 5(6)-carboxy-2′,7′-dichlorodihydrofluorescein diacetate
CNS: Central nervous system
DHR123: Dihydrorhodamine 123
DVL2: Dishevelled segment polarity protein 2
FRET_eff_: Fluorescence resonance energy transfer efficiency
GFAP: Glial fibrillary acidic protein
H_2_O_2_: Hydrogen peroxide
LiCl: Lithium chloride
MYC: v-myc avian myelocytomatosis viral oncogene homolog
NAC: *N*-acetyl-*L*-cysteine
NPCs: Neural progenitor cells
NXN: Nucleoredoxin
PMA: Phorbol 12-myristate 13-acetate
ROS: Reactive oxygen species
RPL13A: Ribosomal protein L13a
RuR: Ruthenium red
TUBB3: Tubulin, beta 3 class III
