# Supplementary figures and images for: Ascorbic acid alters cell fate commitment of human neural progenitors in a WNT/β-catenin/ROS signaling dependent manner
Source: J Biomed Sci. 2017 Oct 16;24:78. doi: 10.1186/s12929-017-0385-1 (PMC5641995; doi:10.1186/s12929-017-0385-1)

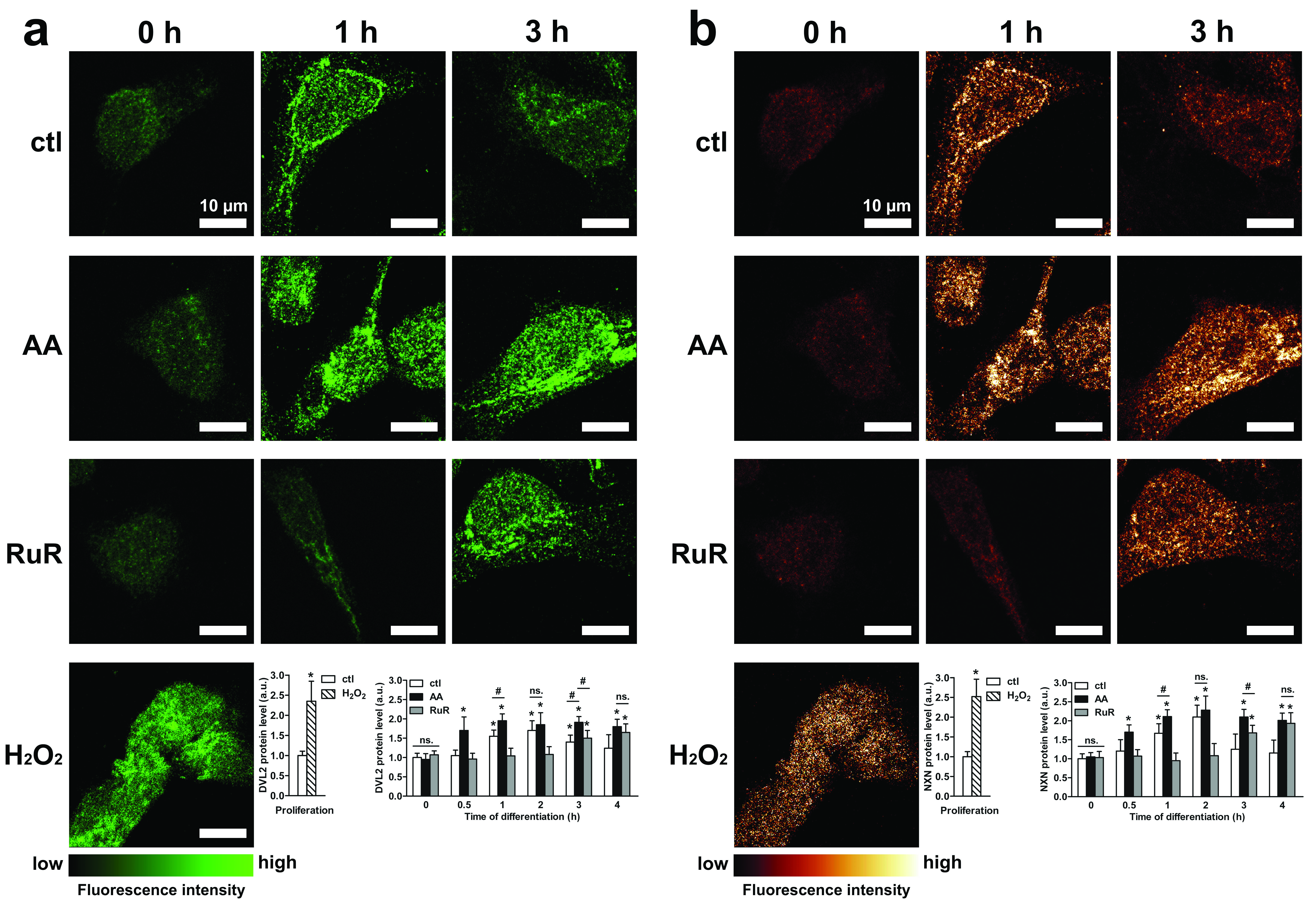

Supplement: Supplementary file 1 — Changes in DVL2 and NXN protein amounts do not correlate with variations in FRETeff. Confocal images of DVL2 (a) and NXN proteins (b) were acquired in proliferating and differentiating cells treated or not with 200 μM AA, 0.5 μM RuR or 1 mM H2O2. Mean fluorescence intensities were then quantified at 0 h, 0.5 h, 1 h, 2 h, 3 h and 4 h of differentiation for each protein (see respective bar graphs). These data demonstrate that decreases in FRETeff values (see Fig. 7) do not result from any reduction in protein amounts which rather increase when DVL2-NXN complexes begin to dissociate. Scale: 10 μm. n = ~ 50 cells per time point and condition. Values are means ± SD of three independent experiments. *P ≤ 0.05 compared with untreated differentiating cells at t = 0 h; # P ≤ 0.05 each treatment condition at each time point; ns, non-significant. (JPEG 5532 kb) [file 12929_2017_385_MOESM1_ESM.jpg]
